# Adapting the Flexible Farrington Algorithm for daily situational awareness and alert system to support public health decision-making during the SARS-CoV-2 epidemic in England

**DOI:** 10.1017/S0950268825000160

**Published:** 2025-02-06

**Authors:** Ian Simms, André Charlett, Felipe J Colón-González, Paula B. Blomquist, Iain R. Lake, Asad Zaidi, Stephanie Shadwell, James Sedgwick, Karthik Paranthaman, Roberto Vivancos

**Affiliations:** 1 Health Protection Operations, UK Health Security Agency, London, UK; 2 National Institute for Health and Care Research Health Protection Research Unit (NIHR HPRU) in Modelling and Health Economics, London, UK; 3 NIHR HPRU in Behavioural Science and Evaluation, Bristol, UK; 4 NIHR HPRU in Vaccines and Immunisation, London, UK; 5 School of Environmental Sciences, University of East Anglia, Norwich, UK; 6 NIHR HPRU in Emergency Preparedness and Response, London, UK; 7 NIHR HPRU in Gastrointestinal Infections, Liverpool, UK; 8 NIHR HPRU in Emerging and Zoonotic Infections, Liverpool, UK

**Keywords:** COVID-19, exceedance algorithm, flexible Farrington, outlier, quasi-Poisson regression, SARS-CoV-2, statistical surveillance

## Abstract

The Flexible Farrington Algorithm (FFA) is widely used to detect infectious disease outbreaks at national/regional levels on a weekly basis. The rapid spread of SARS-CoV-2 alongside the speed at which diagnostic and public health interventions were introduced made the FFA of limited use. We describe how the methodology was adapted to provide a daily alert system to support local health protection teams (HPTs) working in the 316 English lower-tier local authorities. To minimize the impact of a rapidly changing epidemiological situation, the FFA was altered to use 8 weeks of data. The adapted algorithm was based on reported positive counts using total tests as an offset. Performance was assessed using the root mean square error (RMSE) over a period. Graphical reports were sent to local teams enabling targeted public health action. From 1 July 2020, results were routinely reported. Adaptions accommodated the impact on reporting because of changes in diagnostic strategy (introduction of lateral flow devices). RMSE values were relatively small compared to observed counts, increased during periods of increased reporting, and were relatively higher in the northern and western areas of the country. The exceedance reports were well received. This presentation should be considered as a successful proof-of-concept.

## Key findings

- The development of the Adapted Flexible Farrington Algorithm (AFFA) enabled the calculation of daily exceedance thresholds for each of the 316 English Lower-Tier Local Authorities (LTLA).
- Estimates were used to produce visualizations of local COVID-19 case reports and trends.
- The system-generated alerts and local epidemic profiles were a key part of the management of the SARS-CoV-2 epidemic which were used to guide local intervention and control priorities.
- Red–amber–green (RAG) ratings were used to assist interpretation for the local public health teams and, at the national level, in the development of government strategic priorities.
- Retrospective assessment demonstrated that the adapted algorithm performed satisfactorily. The project was considered to be a successful proof-of-concept development.

## Introduction

**Figure 1.**
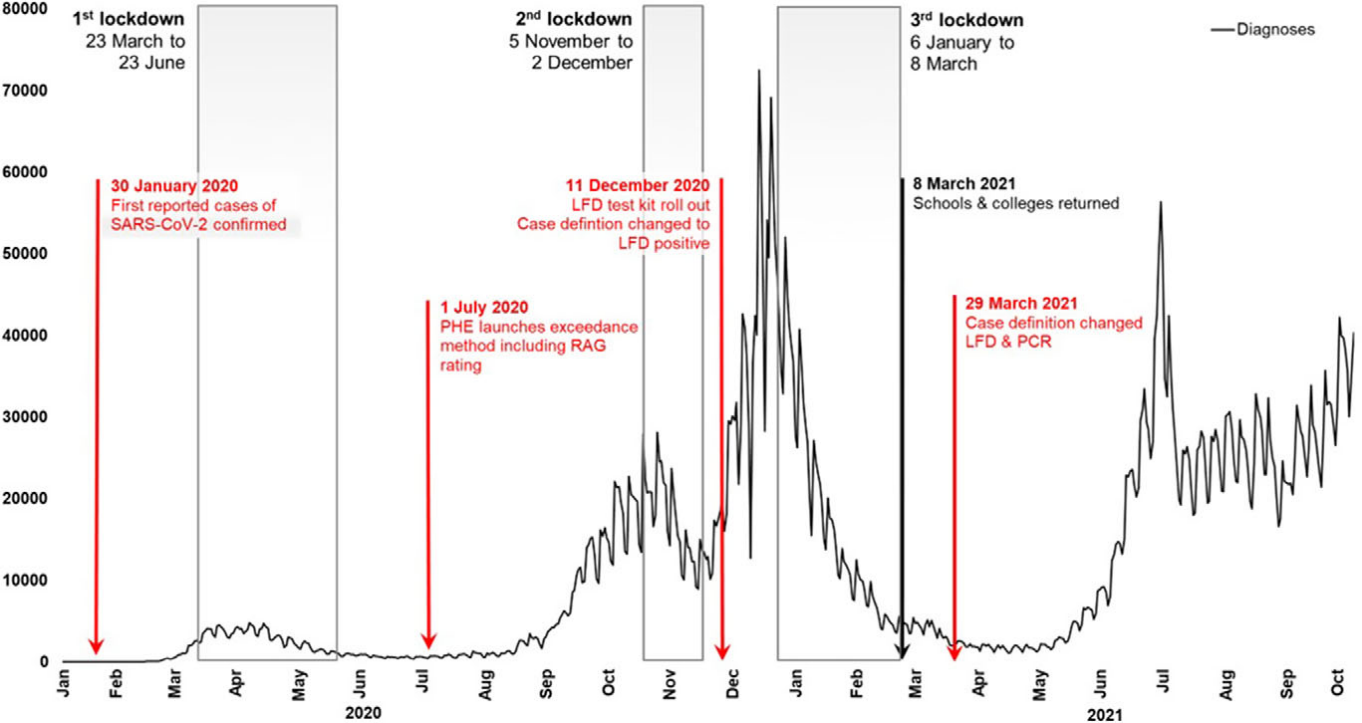
Diagnoses of SARS-CoV-2 and key events in the response to the epidemic, England: 21 January 2020 to 18 October 2021. *Data source:* Public Health England, Second Generation Surveillance System (SGSS) (16).

Globally, the severe acute respiratory syndrome coronavirus 2 (SARS-CoV-2) epidemic was the largest public health emergency to which many public health agencies had to respond [1]. By July 2023, SARS-CoV-2 had led to over 750 million reported coronavirus disease (COVID-19) cases and 4 million deaths [2]. In the UK, the first two laboratory-confirmed cases of the SARS-CoV-2 epidemic were reported on 30 January 2020. Across the course of the pandemic public health systems needed to adapt to the rapidly changing epidemiology of COVID-19. Of particular concern was the rapid emergence of COVID-19 variants and the reported case doubling time which in late 2021 was estimated to be 1.5 to 3 days in England [3]. Initial control measures focussed on non-pharmaceutical interventions, such as social distancing, and latterly relied upon pharmaceutical interventions, such as vaccinations and antivirals [4]. In the UK, the implementation of non-pharmaceutical interventions, such as national lockdowns, changed the course of the pandemic (Figure 1). Once available, pharmaceutical interventions were rolled out. For example, in March 2021, over 3 million people were vaccinated against COVID-19 in 1 week [5]. Diagnostic strategies were also developed rapidly, and in the same month, lateral flow devices became widely available. Initially, COVID-19 case data was only available for hospital and symptomatic testing for high-risk settings but by April 2020 expanded microbiological data became available from wider community testing. To guide local control strategies within this rapidly evolving epidemiological landscape, near real-time (daily) situational and outbreak reports were needed. A system for monitoring disease trends that was less dependent on long-term historical trends was also required.

Before the pandemic, many public health institutions operated automated statistical systems using health surveillance data to monitor disease trends and detect outbreaks. These included the Early Aberration Reporting System (EARS) [6], Early Notification of Community-based Epidemics (ESSENCE) [7], SaTScan [8], and the FFA [9, 10] which is used by many European public health institutions. The UK Health Security Agency (UKHSA) implemented the original Farrington Algorithm in the early 1990s for use on infectious disease surveillance data and now routinely runs the FFA weekly [11–13]. In 2016, the FFA was evaluated using simulated situations considered likely to be encountered within the UK [11]. A coronavirus pandemic was not an anticipated scenario.

Effective communication with all stakeholders involved in the public health response was a key component of the public health response. An early criticism of the UK COVID-19 response was poor communication with local government [14]. Designing timely insightful daily surveillance reports that provided information to support the work of local infection prevention and control teams was a particular challenge.

The adapted Farrington Flexible algorithm (AFFA) provided exceedances of aggregated case reports in precisely the same manner as the FFA, that is, it estimates an upper threshold using results obtained from a quasi-Poisson time-series model. The AFFA was adapted to use a shorter time series of daily counts with cyclical patterns constructed to capture the changes in reporting patterns across the days of the week. The major change from the FFA was that, rather than just considering a single time period as is the case with FFA, we forecast the expected values from the model and constructed thresholds for each of the most recent 14 days. However, unlike the FFA, we did not construct exceedance scores, that is observed minus expected divided by threshold minus expected. This metric was used in the FFA to aid interpretation and is provided to those undertaking outbreak risk assessments using exceedance algorithms. The AFFA produced a simpler aid to interpretation that could be used by local public health professionals who are usually not as familiar with outbreak detection methodologies as infectious disease epidemiologists based in national public health institutes. This consisted of a heuristic RAG rating (see Methods). Both the FFA and the AFFA produce estimates of exponential trends. These would not usually be reported for the FFA because the log-linear time parameter is included within the statistical model to capture longer-term trends due to ascertainment and reporting practices. However, for the short time series used in the AFFA, these growth estimates were provided as a daily incidence rate ratio in addition to the RAG rating. This is because, for short time series, exponential growth estimates are more likely to reflect underlying epidemiology and would therefore be useful in providing an indicator of when local upsurges were occurring.

Here, we describe and assess how the FFA was adapted to the dynamic epidemiological context of the COVID-19 pandemic so that it was capable of supplementing investigator-based surveillance methods to inform public health action within each of the 316 English Lower Tier Local Authorities (LTLA; administrative areas with an average size of just over 180,000 residents).

## Methods

### *Flexible Farrington Algorithm (FFA*)

The FFA is the automated statistical system for health surveillance data implemented within UKHSA. Briefly, a quasi-Poisson regression-based model is fitted to weekly disease counts, with mean (expected count) *μ_i_* and variance *ϕμ_i_* at week *ti.* To estimate disease in the current week, the model is fitted to the previous 5 years of data and includes a linear trend 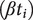 and a yearly 10-level factor 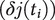 to capture seasonal and sub-seasonal cyclical patterns. The corresponding log-linear model is
(1)

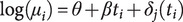

where *j(t_i_)* is the seasonal factor level for week *t_i_*, with *j(t_0_) = 0* and *δ_0_ = 0.* Weeks are highlighted as being possible outbreaks based on the exceedance score:
(2)

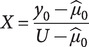

where 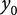 is the current observed count and 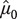 = 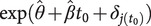 is the current expected count, 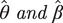 being the respective estimates of 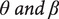 from Equation (1). U, the upper threshold, is the 100(1-α)% negative binomial quantile, α being the type I error probability and set as 0.005. Alarms are flagged for weeks where X ≥ 1. The effect of baseline outbreaks on current predictions is reduced through reweighting of baseline data. The baseline at week *ti* is down-weighted by the inverse of the squared Anscombe residual when the latter is greater than 2.58 [13].

### Adapted Flexible Farrington Algorithm (AFFA)

During COVID-19, the rapidly changing epidemiological situation necessitated the distribution of daily situational and outbreak reports and a system for detecting disease outbreaks that was trained on short term as opposed to long-term historical data. The FFA was adapted accordingly. FFA was chosen as a widely used algorithm, with favourable performance characteristics [15] and was adapted as follows. The AFFA used daily count data and incorporated a day-of-the-week effect rather than calendar periods, and the baseline period of 42 days to remove the need to account for longer-term changes in testing policy. Specifically, the quasi-Poisson regression-based model was fitted to daily counts of reported positive tests, with estimated mean (expected count) 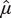 *_i_* and variance *ϕμ_i_* at day *d_i_.* To estimate disease in any one day, the model was fitted to a baseline period (42 days, 15–56 days previously) and included a linear trend and a daily seven-level categorical. The corresponding log-linear model is
(3)

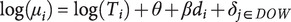

where 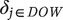 is a seven-level categorical variable for each day of the week, and 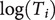 is the offset of the total number of reported tests which was initially included to normalize variations in testing but later dropped from the model (described later in paper). Daily exceedance scores were calculated. Reweighting of outliers in the baseline occurs as per the original FFA [13].

From 1 July 2020, SARS-CoV-2 PCR test results (positive and negative) were extracted from UKHSA databases [16] and used within the AFFA. The primary outcome of interest was PCR-positive SARS-CoV-2 cases by specimen date. The natural logarithm of the number of individuals newly tested was fitted as an offset (included in the model with a coefficient fixed at = 1) to provide estimates of positivity. The delay from the earliest specimen collection date to when test results were reported and available for inclusion in analyses was short, typically 2 days, with around 90% of cases reported by the fourth day after the specimen was taken. The days for which incomplete reporting was expected were highlighted in the time-series graphs provided to local stakeholders to aid interpretation.

### Public health dissemination

The outputs from the AFFA provided key information on subnational (LTLA) trends and local upsurges in COVID-19 cases. To guide local control strategies, clear and effective communication of information was essential. Daily graphs were produced which presented the key data from the AFFA. These resources were developed through an iterative process.

On these graphs, the observed case rate of COVID-19 in the previous 56 days was plotted starting 2 days previously. For example, on 18 March, the graphs would show data for the previous 42 days starting 16 March. Rather than attempting to adjust for reporting delays, the current and previous days are not presented as the surveillance data was largely incomplete. Data for the previous 56 days were subdivided into the period of interest (previous 14 days) and the baseline period (15 to 56 days previously). From the AFFA, the expected case positivity was also presented alongside the upper 99% exceedance threshold. Days exceeding the 99% threshold were labelled, and the graphs also presented the number of people tested each day for COVID-19. Due to reporting delays, the last 4 days of case numbers were likely to be somewhat incomplete, and hence, these data were labelled as uncertain.

To provide a readily interpretable summary, a red–amber–green (RAG) rating was used to provide a daily classification for each LTLA. A RED rating is given if the threshold is exceeded for two or more of the 14 most recent days regardless of the magnitude of the exceedances, or if the observed cases are greater than the expected value for 12 of the 14 most recent days. An AMBER rating was given if the threshold is exceeded for only one of the 14 most recent days, or if the observed cases are greater than the expected value for 10 or more of the 14 most recent days. Otherwise, a GREEN rating was given. In addition, for each LTLA, further detail was produced including temporal trends and the geospatial distribution of disease burden by gender, risk group, and healthcare setting [17].

### Assessment of the AFFA

An assessment of the AFFA was conducted based on 300 days of model outputs between 18 March 2021 and 11 May 2022 for each of the 316 LTLAs. This period is longer than 300 days while in the earlier period of the pandemic results from the AFFA were provided daily including weekends. Over a 300-day period, the 42-day baseline case data together with the model predictions for the period of interest were used. The ideal performance characteristic metrics of detection sensitivity and specificity and timeliness of detection [15] could not be estimated due to the lack of knowledge of when and where true periods of increased incidence occurred. Since the AFFA provided an assessment of how close the observed reported case numbers were from the model expectation, and ‘flagging’ situations where these exceeded the upper 99.5% threshold, it was reasonable to use the root mean square error (RMSE) of the forecasts to assess the algorithm.
(4)

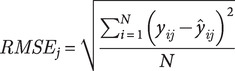

This was calculated for each of the 14-day prediction periods 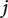 , combining data across the 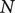 LTLAs for each run of the algorithm over the 300 days, providing a means of assessing by how much the observed reported cases 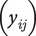 diverge from the algorithms forecast 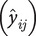 . Spatial variability in the RMSE for the 1 day ahead forecast was explored. The RMSE, calculated for each LTLA, pooling data from all the model runs. Finally, to explore how the RMSE varied over time, estimates for each day were obtained from data across all LTLAs.

## Results

### Public health dissemination

**Figure 2.**
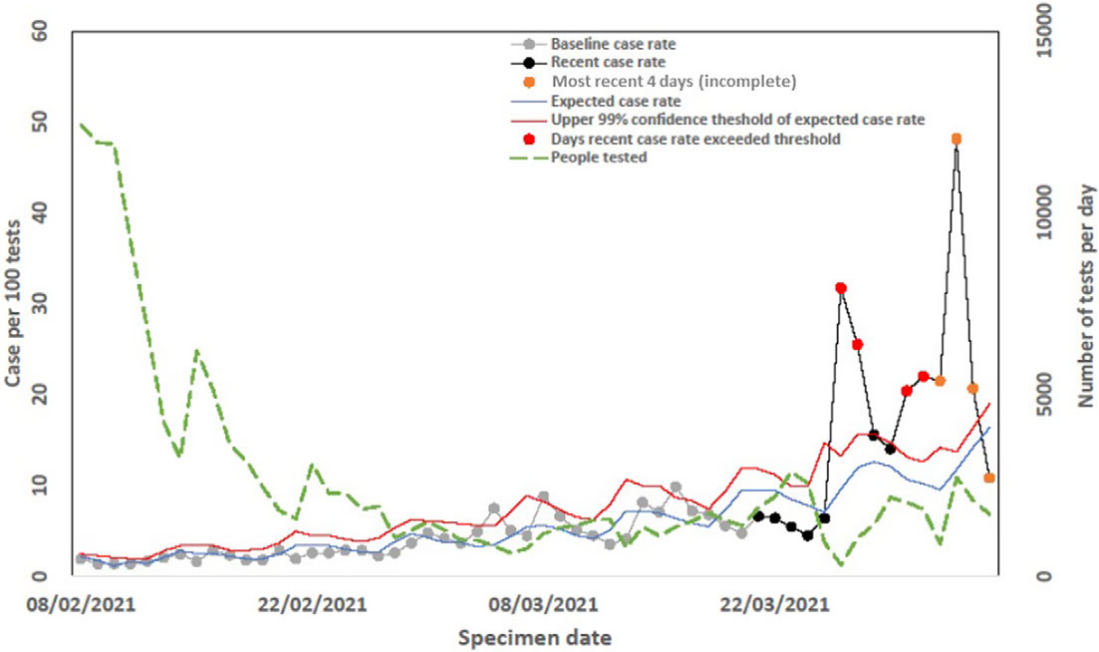
Daily SARS-CoV-2 exceedance report published 5 January 2021 for one LTLA.

The AFFA was successfully implemented, and daily outputs were produced for LTLAs from 1 July 2020. A sample output for one LTLA shows the modelled pattern of reports over the days of the week (expected case rate (blue) and the upper 99% threshold (red) (Figure 2). This demonstrates how the AFFA has accounted for day-of-the-week effects. On the line for the recent case rate (black), days exceeding the upper 99% confidence threshold are labelled red and the most recent 4 days are labelled orange to alert for potential impacts of reporting delays. Based on the criteria given in Table 1, this LTLA would have a red RAG rating, that is upper threshold exceeded for 7 of the 14 most recent days, indicating further investigation was warranted by local health protection teams.

**Table 1.** Definition of red–amber–green (RAG) rating

Classification	Definition
Red	Upper threshold was exceeded for ≥2 of the 14 most recent days regardless of the magnitude of the exceedances, *or* if the observed case count was greater than the expected case count for ≥12 of the 14 most recent days.
Amber	Upper threshold was exceeded for 1 of the 14 most recent days, *or* if the observed case count was greater than the expected case count for 10 or 11 of the 14 most recent days.
Green	Upper threshold was not exceeded during the most recent 14 days *and* the observed case count was greater than the expected case count on ≤9 of the most recent 14 days.

### Modifications to the AFFA

**Figure 3.**
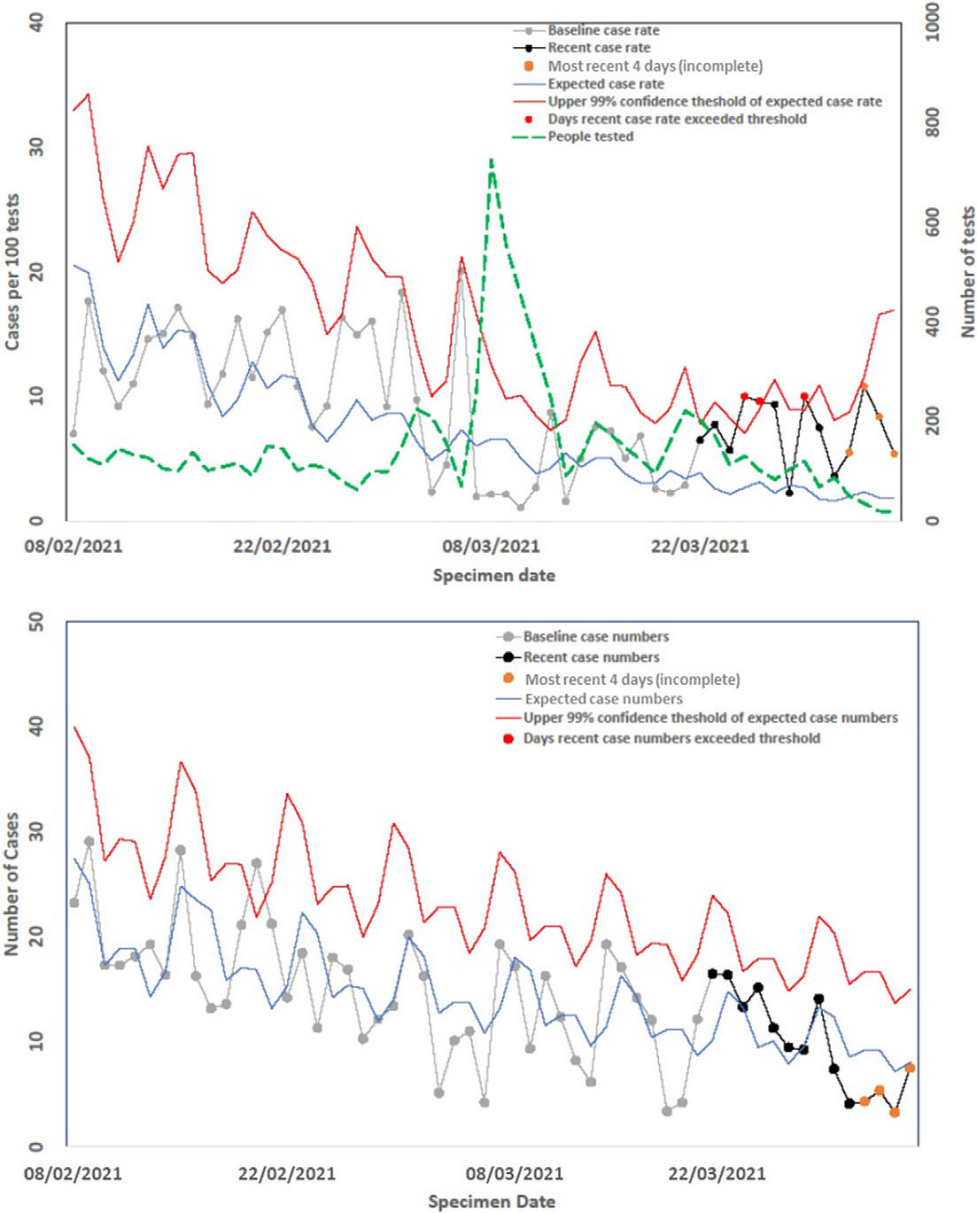
Influence of rapid expansion of LFD testing on AFFA published 6 April 2021 for one LTLA. (a) Model based on case numbers but offset by testing volume. (b) Model based on case numbers only.

Modifications were required due to the rapid expansion in testing facilitated using lateral flow devices (LFDs). From 8 March 2021, the use of LFD expanded rapidly as secondary school children began regular testing as part of the government’s strategy to reopen schools [18]. Using data from a different LTLA, the impact of this modification is seen as a testing spike (green dashed line) around 8 March 2021 (Figure 3a). Initially, the greater amount of testing increased the offset in the model which effectively reduced the positivity. However, around 1 week later, as the time window moved forward and the spike in testing volumes fell, a higher proportion of LTLAs were classed with a red RAG. This is because the reduction in testing effectively increased the positivity, most likely as a result of decreases in the volume of unpremeditated LFD testing. The large variations in testing numbers were inducing spurious variations in positivity. This artefact was expected to continue and, since case numbers were the primary public health focus, the AFFA was adapted so that it was based solely on reported case numbers. This was achieved by removing the model offset. The impact of this change is illustrated in Figure 3b for the same LTLA and shows that recent cases are now below the upper threshold and that the LTLA RAG rating LTLA has changed from red (Figure 3a) to green (Figure 3b).

### Assessment of the AFFA

The AFFA was assessed in three steps. Firstly, the x-days ahead prediction RMSE was explored to assess how these varied the further into the future the model predictions are being made. Across all LTLA’s, the prediction 1 day into the future had a mean RMSE of 39.7 cases per day (95% CI 36.8–42.6). As anticipated, this mean was 2.5 times higher when produced for 14 days into the future (102.8 cases per day, 95% CI 94.3–111.3). For context, the mean number of cases across all LTLAs was 216 per day with a median value of 161 cases per day.

**Figure 4.**
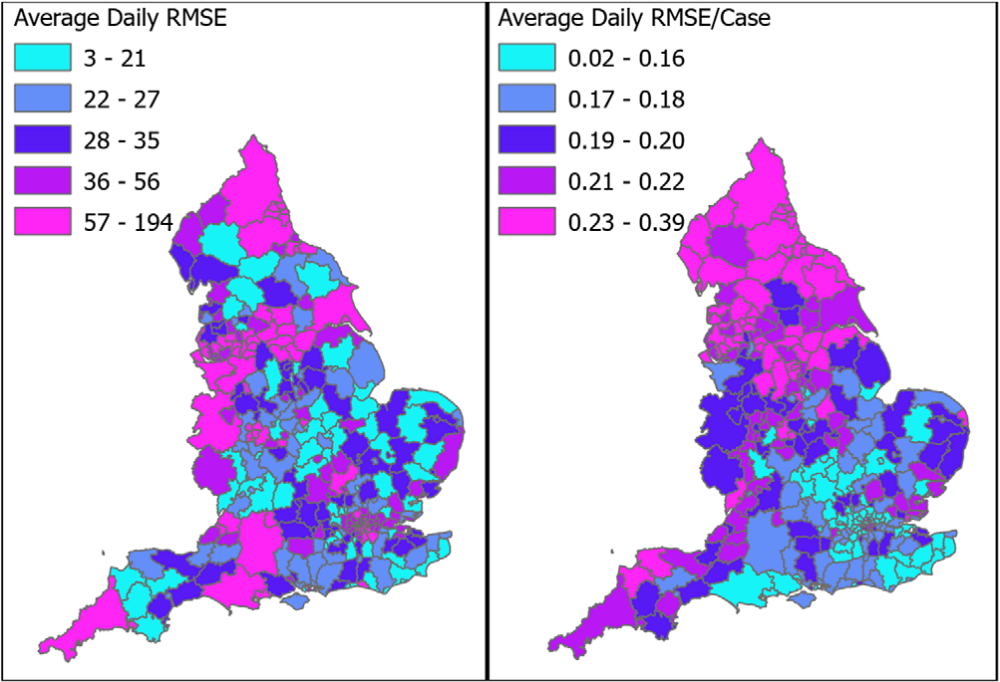
RMSE (a) and RMSE/case (b) across all LTLA’s in England for predictions 1 day into the future.

To explore whether there was evidence that AFFA might be performing differently in certain parts of the country, the mean RMSE one day into the future, and the RMSE divided by average daily case numbers for each LTLA were mapped (Figure 4).

Some spatial variation in the RMSE and RMSE/cases can be seen in Figures 4a and 4b. These were relatively higher in the northern and western parts of the country. While this extra variance would be taken into account in setting the AFFA thresholds, its likely impact would be to lower the detection sensitivity and increase the time to detection of periods of increased incidence in the LTLAs in these parts of the country.

**Figure 5.**
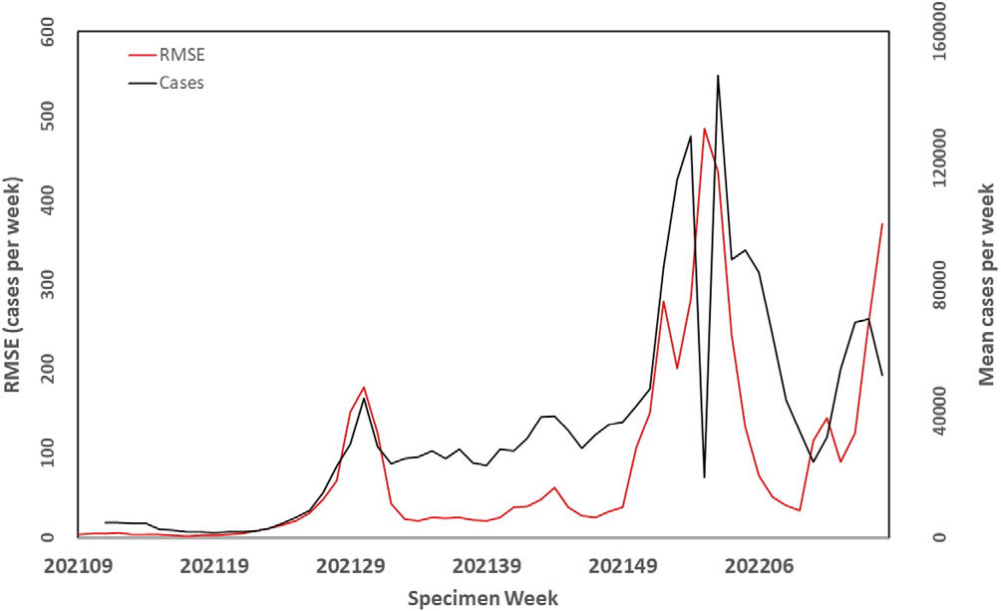
Time-series plot of COVID-19 case numbers in England and the RMSE from the prediction made 1 day into the future using the Adapted Flexible Farrington Algorithm.

Finally, the temporal pattern in the RMSE was assessed. Case numbers were plotted against the RMSE (1 day into the future) averaged over all LTLAs and weeks (Figure 5). The figure shows that when incidence was low in early 2021, the RMSE per case was small, but after this period when incidence increased, the RMSE also increased indicating that the reported counts and the algorithm predictions were diverging. This is precisely what you would want to occur to enable the algorithm to ‘flag’ that there is a rapidly increasing incidence in reported cases. As observed in most infectious disease surveillance systems, a large decrease in reported cases occurred at Christmas (Specimen Weeks 51/52).

## Discussion

Here, we demonstrate how the development of the AFFA enabled the calculation of daily exceedance estimates for each English LTLA. We have further highlighted how these estimates were used to produce visualizations of local COVID-19 case reports and trends and used to provide an RAG rating to assist interpretation for the local public health teams. These system-generated alerts and local epidemic profiles were a key part of the management of the SARS-CoV-2 epidemic which were used to guide local intervention and control priorities [17]. For example, in the late summer of 2020, LTLA’s with a red RAG rating were prioritized to receive additional resources focussed on testing for COVID-19 and tracing case contacts. RAG ratings were also used at the national level in the development of government strategic priorities. When the testing policy changed, there was a greater increase in the ascertainment of negative test results compared to that observed for reported positive test results. Such time-series ‘shocks’ were clear and obvious when accounting for testing volumes using test positivity. These ‘shocks’ impacted the total test positive reports, but their magnitude appeared less obvious to discern. The inclusion of additional predictor variables within the time-series model could have been used to explore the volatility within the reporting process, but we were unable to address this as time and resources were at a premium during the pandemic.

Many automated statistical systems of health surveillance data work using weekly surveillance data [19], and some work with daily data [20]. Interest in daily systems has developed for two reasons. Increased analytical frequency offers the opportunity to detect and control incidents earlier. The data collection process also provides benefits in terms of situational awareness of developing incidents or reassurance that an incident has not developed [21]. Statistical summaries of case reporting cannot be used alone to guide local control strategies. However, they do assist in providing insight into whether current case numbers are deviating from extrapolated trends and the trend estimates themselves indicate whether there are changes in the underlying incidence within the population. All the statistical information from the AFFA together with local situational awareness such as clusters of cases in schools and workplaces, and spatial distribution of cases, provided local public health professions with a consolidated view of how SARS-CoV-2 was transmitted within their locality. This allowed local risk assessments to be undertaken to assist in determining whether any additional public health action was warranted.

The degree to which the AFFA and RAG ratings were accepted and used during COVID-19 was an important indication of their validity. The speed with which the AFFA was implemented without a thorough evaluation suggests that the AFFA and subsequent RAG ratings presented in this paper should be viewed as a proof-of-concept development. The daily AFFA is an adaptation of the weekly FFA which itself is recognized for its high sensitivity, specificity, and positive predictive value [15]. The preliminary assessment of the algorithm presented provides reassurance that the AFFA is performing as anticipated. However, further research and evaluation are needed to explore refinements to the algorithm. For example, the optimal length of the baseline period, the inclusion of non-linear trends and offset specification, using synthetic with known periods with varying increased incidence to better understand the performance characteristics of the AFFA to detect outbreaks and investigate how this compares to other algorithms [13, 15]. We would contend that the RMSE can be used as a method of providing a rough guide as to whether the algorithm provides results that can be used for public health action when considered together with other local intelligence.

To enhance the validity of the AFFA, future research needs to consider its ability to work beyond COVID-19. COVID-19 incidence was relatively high and additional challenges may emerge for less common diseases especially at a local level. For example, in the early stages of the epidemic, changes in testing strategies led to artefacts in the comparison of local epidemic trajectories using the AFFA. The more stable testing volumes later in the pandemic minimized these issues, although technical challenges emerged due to the increasingly large quantities of data needing to be processed.

One advantage of the AFFA was that it was not fully automated. This allowed modifications to be made in response to operational requirements and ensured that accurate, easily accessible information was produced to support local decision-making. For example, early in the development process, an initial modification was made to control the proportion of false alarms without compromising the detection of genuine alarms. All changes were tested in parallel with ‘live’ surveillance processes and implemented after consultation with the UKHSA Incident Director and LTLAs.

The validity of the graphical outputs from the AFFA and the associated RAG ratings was enhanced by the degree to which it was used during COVID-19. The RAG ratings were chosen as they are commonly used for status reporting within public health [22]. In addition, surveys of the SARS-CoV-2 surveillance outputs produced by the UKHSA were generally viewed favourably by local stakeholders [17]. Yet the speed of implementation prevented a thorough inclusion of user perspectives at the outset which is key to the success of such information [23]. Further development of outputs through a user consultation involving the wide audience of the AFFA outputs would be useful. This would be particularly important when the format of the outputs needs to be altered. For example, in March 2021, the removal of the offset term from the AFFA effectively changed the analysis from positivity to case reports (Figure 3a vs. 3b). Although this statistical change was implemented quickly, it took longer to address user concerns. These related to the effect that the removal of the denominator had on the interpretation of the graphs and statistical outputs in relation to drivers within the epidemic at national and LTLA levels.

## Conclusions

We anticipate that our experience with the AFFA and the development of graphical and statistical outputs at the local level will motivate more thorough evaluations of the AFFA and the outputs leading to similar systems in other settings.

## Data Availability

The anonymized datasets used in our study are confidential records supplied to UK Health Security Agency under Regulation 3 of The Health Service (Control of Patient Information) Regulations 2020 and under Sect. 251 of the NHS Act 2006. In accordance with the UKHSAs duty of confidentiality and associated legal restrictions, the datasets analyzed during the current study are available from the corresponding author on reasonable request.
